# Current status of undergraduate teaching in forensic & legal medicine in Europe

**DOI:** 10.1007/s00414-024-03224-0

**Published:** 2024-04-17

**Authors:** Jason Payne-James, Grace Payne-James, Rossana Cecchi, Denis Cusack, Eva Keller, Bertrand Ludes, Burkhard Madea, Marika Väli, Duarte Nuno Vieira, Antti Sajantila

**Affiliations:** 1 Medical Examiner Service, Norfolk and Norwich University Hospital, Colney Lane, Norwich, NR4 7UY UK; 2 Forensic Healthcare Services Ltd, Southminster, CM0 7DT UK; 3 Department of Forensic Medicine, University of Helsinki, PO BOX 21 (Haartmaninkatu 3), 00014 Helsinki, Finland; 4 Forensic Medicine Unit, Finnish Institute for Health and Welfare, Helsinki, Finland; 5 Unit of Legal Medicine, Department of Biomedical, Metabolic and Neural Sciences, University of Modena and Reggio Emilia, Modena, Italy; 6 Forensic and Legal Medicine and Medical Bureau of Road Safety, University College Dublin, Ireland and Coroner Service of Ireland, Dublin, Ireland; 7 Faculty of Medicine, Department of Forensic Medicine, University of Debrecen, Debrecen, Hungary; 8 Université de Paris, CNRS, BABEL, F-75012 Paris, France; 9 Institute of Forensic Medicine, University Hospital Bonn, Stiftsplatz 12, 53111 Bonn, Germany; 10 Institute of Pathological Anatomy and Forensic Medicine, University of Tartu, Tartu, Estonia; 11 Institute of Legal and Forensic Medicine, Faculty of Medicine, University of Coimbra, Coimbra, Portugal; 12 European Council of Legal Medicine, London, UK

**Keywords:** Forensic & legal medicine, Legal & forensic medicine, Forensic medicine, Forensic pathology, Undergraduate, Education, European Council of Legal Medicine, ECLM

## Abstract

The European Council of Legal Medicine (ECLM) is the body established in 1992 to represent practitioners forensic & legal medicine and is composed of delegates of the countries of the European Union (EU) and from other countries which form part of Europe to a current total of 34 member countries. The aims of this study were to determine the current status of undergraduate forensic & legal medicine teaching in the curriculum of medical studies in ECLM countries and to use the results of this study to determine whether it would be appropriate to develop new guidelines and standards for harmonising the content of undergraduate forensic medicine training across ECLM member countries. A detailed questionnaire was sent to all individuals or organisations listed on the ECLM contact database. Responses were received from 21 of 33 countries on the database. These responses showed considerable emphasis on undergraduate teaching of forensic medicine in all countries with the exception of Belgium and the United Kingdom. There was great general consistency in the subjects taught. The data from this survey provide a baseline which should assist in developing a strategy to harmonise forensic & legal medicine undergraduate training in member countries of the ECLM. The ECLM is now in a good position to establish a pan-European working group to coordinate a consensus document identifying an appropriate and modern core undergraduate forensic medicine curriculum that can be presented to the medical education authorities in each country, and which can be adapted for local requirements, based on available personnel, the forensic medicine structure in the country, and most importantly, the needs of the local population.

## Introduction

The terms forensic medicine, forensic pathology, legal medicine, forensic & legal medicine and legal & forensic medicine are used interchangeably worldwide. In very broad terms, practitioners in these fields are medical doctors (and other relevant healthcare professionals) who work at the interface of medicine, law and justice systems.

The European Council of Legal Medicine (ECLM) is the body established in 1992 to represent such practitioners and is composed of delegates of the countries of the European Union (EU) and from other countries which form part of Europe [1]. Countries are included as ECLM members provided that within them Legal and Forensic Medicine is either fully recognised as a discipline and/or has an established university academic structure and/or another official structure which the ECLM after due consideration, deems to be of an equivalent standing and currently stands at 34 member countries [2].

The aims and scope of the European Council of Legal Medicine are fourfold and described in the Statutes [3] thus:

1. The ECLM shall be the official body dealing with matters relating to Legal and Forensic Medicine in Europe.
2. The ECLM shall deal with all scientific, educational and professional principles and matters pertaining to this discipline on a European level.
3. The ECLM shall especially pursue the recognition of the discipline and harmonisation of practices and quality assurance in the speciality at European level.
4. The persons professing this discipline shall be medically qualified persons involved in the investigation and assessment of unexpected and/or unnatural deaths, bodily harm or personal injury within the framework of the legal system and/or in the teaching of this speciality.

The ECLM published the ‘Perugia document’ which addressed the teaching of legal medicine to undergraduate medical students in Cologne in July 1992 [4]. The preamble to the document notes:

From the first few days after qualification, the practitioner of medicine is of necessity exposed to tasks and problems during the course of the daily professional duties which require substantial medicolegal knowledge to be dealt with proficiently and satisfactorily. The scope of this document is to produce guidelines of a minimum standard as a basis for the undergraduate curriculum in Legal Medicine. Varying nuances of emphasis would be required in individual countries to take into account differences in local practice, custom and legislation.

Major Curricular Topics

The major topics in Legal Medicine to be highlighted in undergraduate teaching are the following main headings:

1. Thanatology and Forensic Pathology
2. Clinical Forensic Medicine
3. Medical Law and related Jurisprudence.

The nature of forensic medical work has changed and expanded substantially in the last three decades. This change has been driven by social, geopolitical and international needs and themes, including violence against women & girls, female genital mutilation, mass migration and non-fatal strangulation, and by exponentially growing technological advances deriving from basic research in life sciences. Most healthcare professionals, whatever their specialty, whether in primary or secondary care settings will encounter many of the matters embraced by forensic medicine. There has been a concern in some countries that undergraduate forensic medicine teaching is not prioritised within medical training at a time when knowledge about forensic medicine is needed more than ever, particularly as it currently affects more areas of the health care system than in the past. The nature of undergraduate forensic medicine education is variable across Europe but to date there are no data available to identify that variability and to allow consideration of harmonising such teaching.

## Aims

The aims of this study were 1) to determine the current status of undergraduate forensic and legal medicine teaching in the curriculum of medical studies in countries who are members of (or are applying to be) members of the ECLM; and 2) to use the results of this study to determine whether it would be appropriate to develop new guidelines and standards for harmonising the content of undergraduate forensic and legal medicine training across ECLM member countries.

## Methods

A detailed questionnaire in the English language was created on SurveyMonkey and sent electronically to emails of all individuals or organisations listed on the ECLM contact database (*n* = 99). These emails included delegates, deputies, observers and representatives of the country's National Authority for forensic medicine. Because of differences in languages, legal systems and medical training between countries, the following definitions were utilised for this study:

- Europe
  Countries which constitute the membership of the ECLM (defined in the ECLM Statutes as: (1) … countries of the European Union (EU) and from other countries which form part of Europe) [3]. This definition is not necessarily aligned with United Nations or other standardised definitions.
- Legal and forensic medicine
  Varied terminology (including forensic medicine, forensic pathology, forensic & legal medicine, medical jurisprudence and others). ‘Forensic medicine’ was the generic term used in this study.
- Undergraduate
  A student training to become a medical doctor (not other allied health professionals).

The questionnaire addressed matters related to the state of undergraduate forensic medicine teaching in respondents’ own countries. The email providing survey details was sent on 12^th^ June 2023 and to all non-responders again on 15^th^ July 2023. The survey was closed on 24^th^ August 2023. Appendix 1 shows the text of the introductory email. Appendix 2 shows the questions within the survey.

## Results

A total of 45 responses were received to 99 survey requests, a response rate of 45%. A further 18 responses were received as notifications that ‘this email is no longer active’. No response at all was received from 36/99 (36%).

**Fig. 1:**
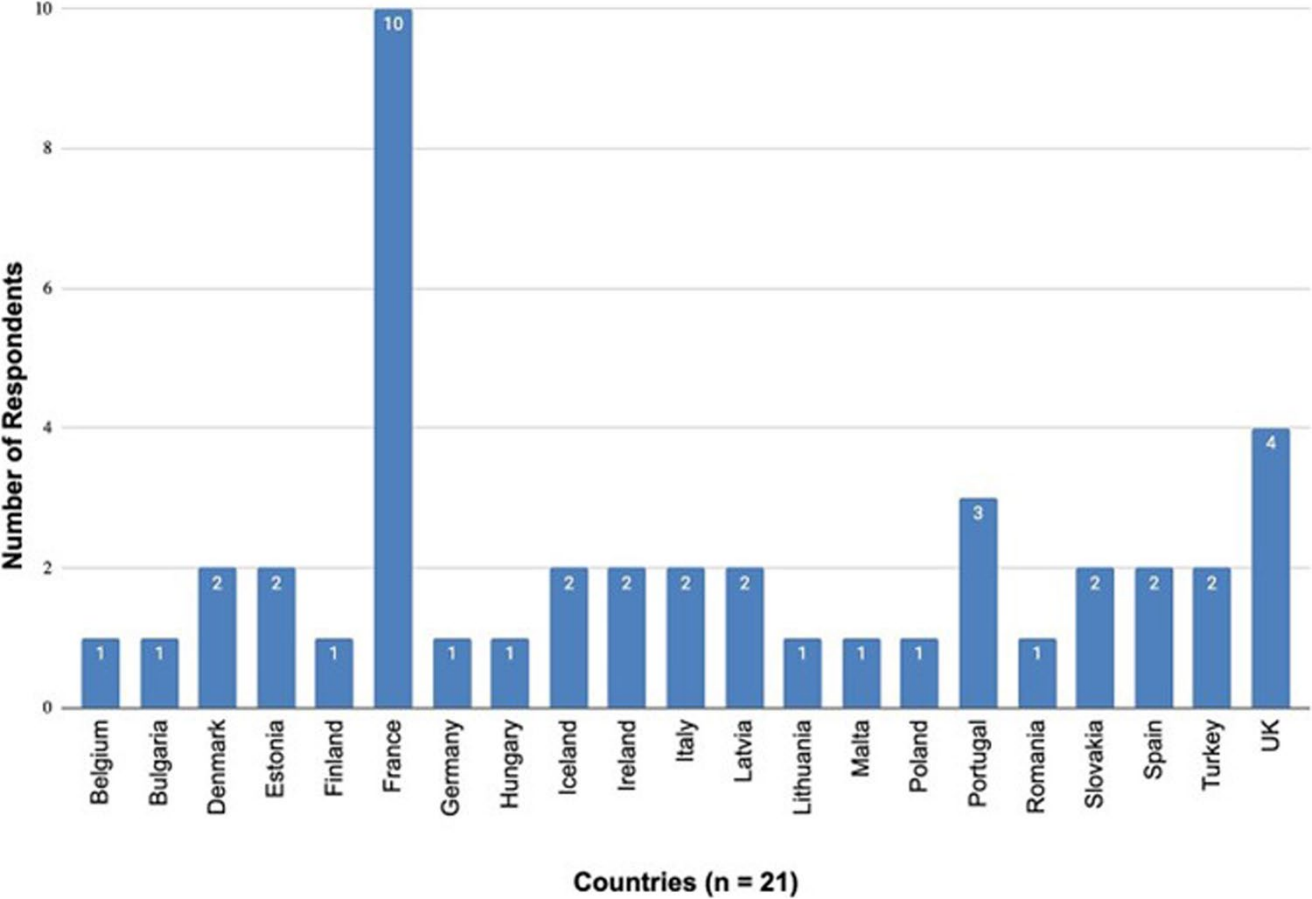
Number of responses by country (45 responses from 21 countries)

Figure 1 shows the number of responses from each of the 21 countries from whom a response was received. As survey questionnaires were sent to all delegates, deputies, and observers to the ECLM or representative of their country's National Authority for forensic medicine, some countries provided more than one completed survey (range 1–10). Linguistic challenges (as the survey was written in the English language) may have resulted in some responses which did not address the question asked. There were typically one to two responses per country (three for Portugal and four for UK), but France clearly stood out with 10 complete or partial responses. Table 1 shows the job titles of the respondents to the survey. From 31 titles, 22 had a a term ‘forensic’ in their titles. Others included references to Professor (Emeritus Professor), and resident, where the respondent most likely is (or has been) directly involved in the forensic field, or specialists in the medical ethics and law or in clinical medicine. Table 2 shows the responses for the number of medical schools in each country. There was a lack of consistency in responses where there was > 1 response from each country. Table 2 also shows the population size as estimated in 2022 [5]. The population embraced by the respondent countries was ~ 536 million. 17 of the 21 (81%) countries indicated that forensic medicine was a compulsory part of the undergraduate medical curriculum in their country. Two respondents (the United Kingdom and Belgium) indicated that it was not compulsory and two countries (France and Portugal) had respondents who gave different responses (in Portugal’s case related to the establishment of private medical schools with non-standard curricula).

**Table 1:** List of job titles of each respondent

Associate Prof of Medical Ethics & Law
Chef de Clinique
Chef de Clinique des Universités—Assistant des Hôpitaux
Clinical Senior Lecturer in Forensic Pathology
Consultant Forensic Physician
Delegate of the ECLM, Professor, Director of the National Institute of Legal Medicine
Doctor
Doctor in Legal Medicine & Medical Expert
Emeritus Professor
Forensic Doctor
Forensic Medical Expert
Forensic Medicine
Forensic Medicine (The State Forensic Medicine Service)
Forensic Pathologist
Forensic Pathologist, Forensic Radiologist, Head of Department
Forensic Practitioner & Neuropathologist
Full Professor
Full Professor—University
Full Professor in Legal Medicine
Head of Department of Forensic Medicine—District Hospital Senior Lecturer in Pathology
Head of the Department of Forensic Medicine
Legal and Forensic Medicine Specialist
Legal Doctor
MD, PhD
Médecin Légiste
Praticien Hospitalier
Prof. Dr Hab. n. Med
Professor (× 3)
Professor Emeritus of Forensic &Legal Medicine, Director of Medical Bureau of Road Safety, and Coroner Service
Professor Forensic Pathology & Forensic Anthropology
Professor of Forensic Medicine
Professor, Chief Forensic Pathologist
Professor, State-Appointed Forensic Pathologist
Resident
Senior Forensic Pathologist, Director of the Forensic Medicine Expertise Department; Lecturer in Forensic Medicine
Specialist in Forensic & Legal Medicine, Professor
Specialist in Forensic Medicine
Specialty Doctor/Forensic Physician

**Table 2:**
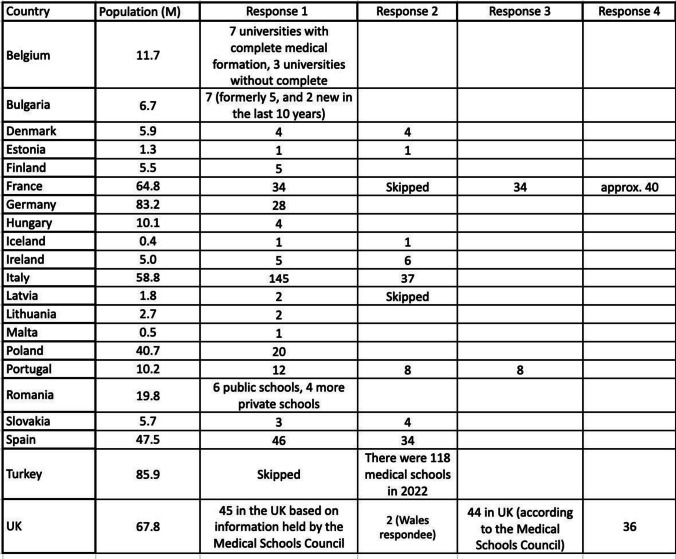
Responses to the question about the number of medical schools in each country. Country population in 2023 provided in millions (M) [5]

The Table only shows up to 4 responses (France had 6 additional responses)

Table 3 shows the response to questions which asked how many hours were allotted to the undergraduate forensic medicine teaching (in those countries where it was compulsory). The range varied from 8 to 250 h. The country with the most amount of teaching was Spain. Some responses (eg from Romania) may have reflected all education (rather than forensic medicine teaching).

**Table 3:**
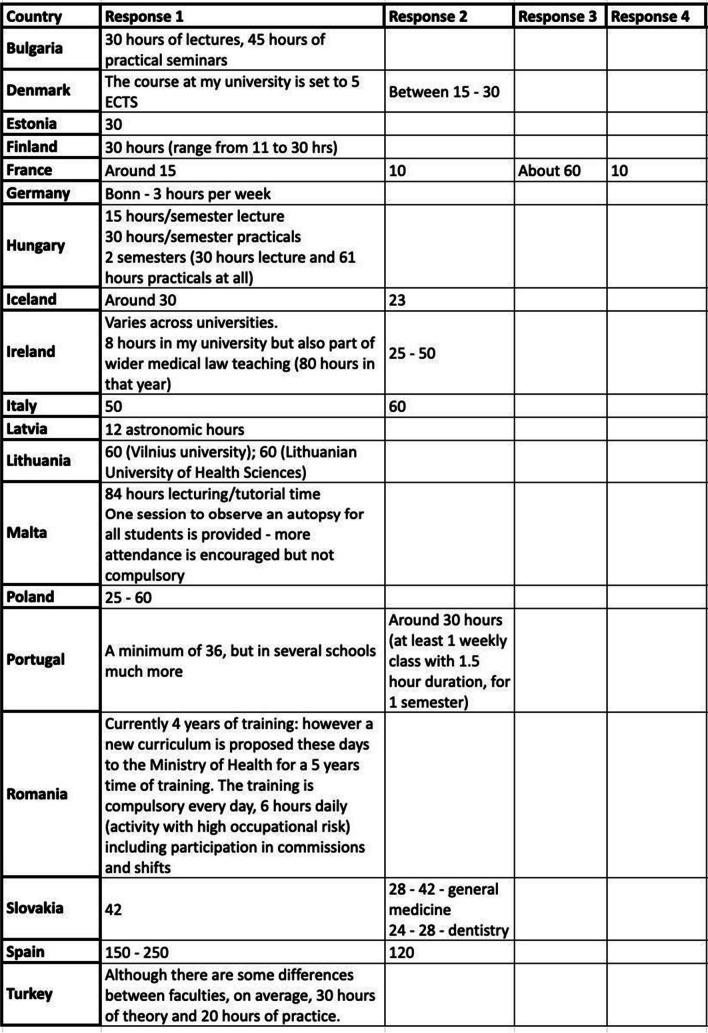
Hours allotted to undergraduate forensic medicine teaching by country

**Fig. 2:**
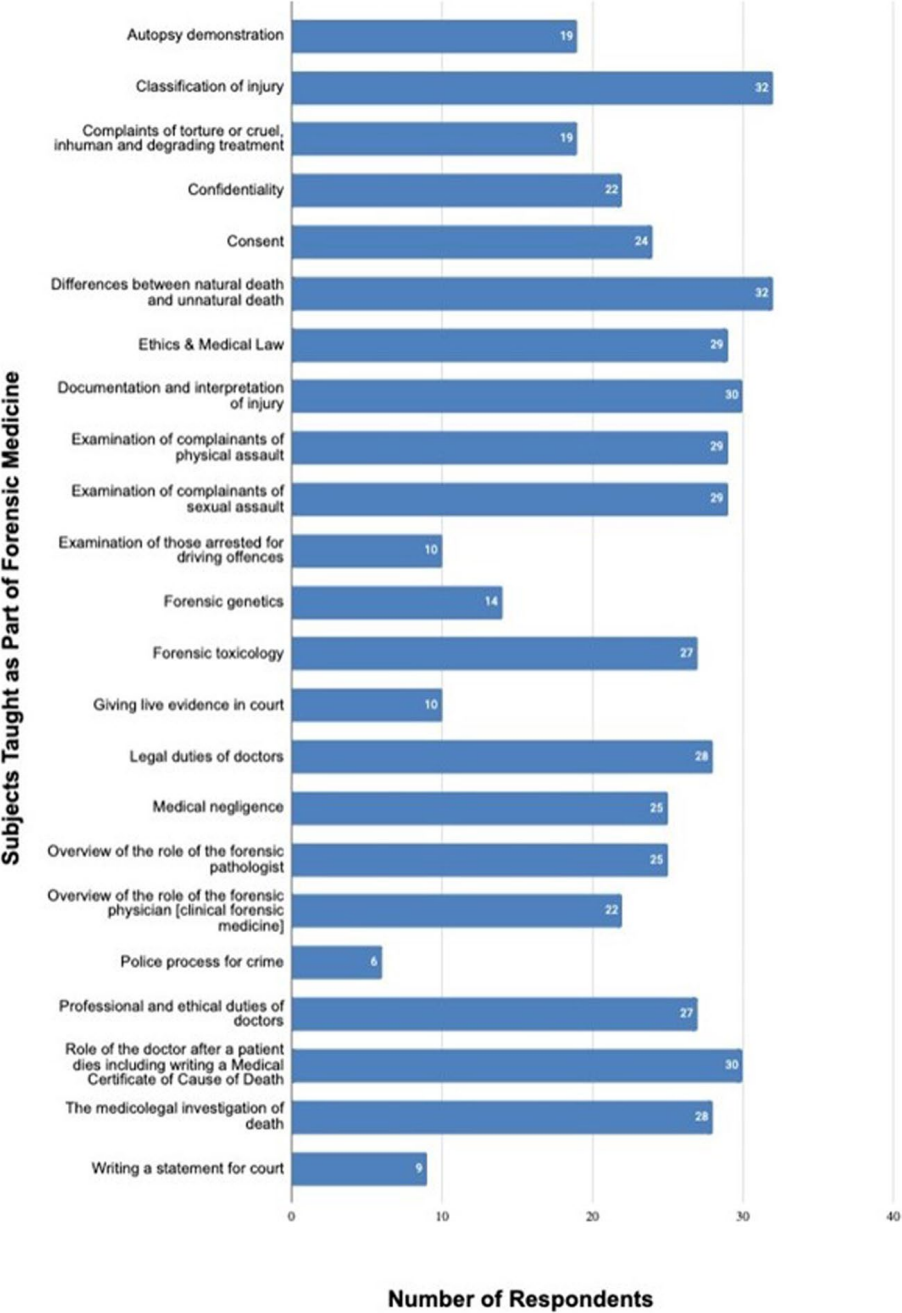
Forensic medicine subjects taught to undergraduate medical students

Respondents were provided with a list of subjects that embraced many forensic medicine subjects, and asked to identify which ones were taught to their forensic medicine graduates. Figure 2 summarises the responses by subject. There was a considerable consensus on what subjects were taught across the respondent countries. The least frequently taught subjects were: a) examination of those arrested for driving offences; b) forensic genetics; c) giving live evidence in court; d) writing a statement for court; and e) the police process for crime. Respondents were asked to provide free text responses identifying additional subjects taught in their country which weren’t listed in the survey. All responses were included from each country as it was considered that respondents who mentioned specific subjects did so from personal knowledge of it being taught.

Table 4 shows the list of additional subjects related to forensic medicine taught in each country. These include e.g. external examination of the body and secondary signs of death (Denmark, Finland, Iceland, Romania) and various themes of evaluation of injury and fitness, and questions of compensation (Italy, Hungary, Slovakia, Portugal).

**Table 4:**
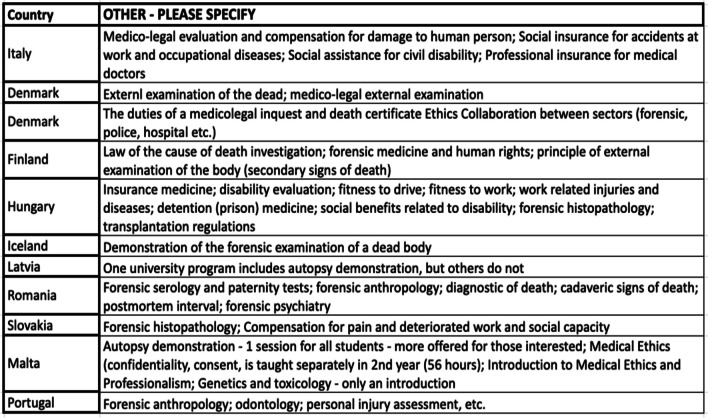
Additional taught subjects in forensic medicine by country (free text responses)

**Fig. 3:**
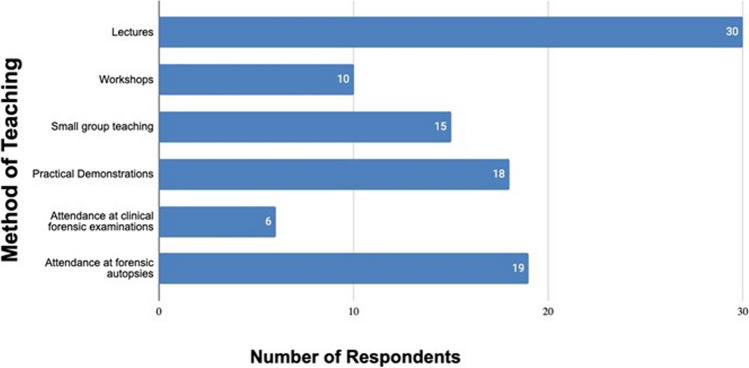
Methods of teaching forensic medicine

Figure 3 shows the teaching methods used. Lectures remain the most common method of teaching with attendance at forensic autopsies (*n* = 19), practical demonstrations (*n* = 18) and small group teaching (*n* = 15) being the other common techniques. Attendance at clinical forensic examinations was the least used modality (*n* = 6). When considered how completion of the forensic medical teaching module was assessed written examination (*n* = 19) and MCQ examination (*n* = 19) were most frequent with a smaller number using oral examination (*n* = 9). Similarly to data in Fig. 2 all responses were included from each country as it was considered that respondents who mentioned specific teaching methods did so from personal knowledge.

The most common materials used to accompany teaching forensic medicine were textbooks (*n* = 29) (the textbooks referred to are listed in Table 5), lecture notes (*n* = 26), and scientific articles (*n* = 19). Fourteen respondents were unaware of the ECLM Perugia document about teaching undergraduate legal medicine [3].

**Table 5:** Textbooks referred to as being used in undergraduate forensic medicine training by a respondents (responses provided as written by respondents)

Medicina Legal y Toxicologia, Villanueva Cañadas
Cazzaniga CM, Cattabeni, Luvoni R, Zoja R. Compendio di Medicina Legale E Delle Assicurazioni, Utet, Torino, 2022
Nordisk Lærebog i Retsmedicin (Nordic Forensic Medicine)—Thomsen et al
Retsmedicin, Jørgen Lange Thomsen. Knight’s Forensic Pathology, 4th ed. Pekka Saukko, Bernard Knight. Lægens Roller, Louise Binow Kjær(red.), FADLs Forlag
Simpson’s Forensic Medicine for English speaking students and other textbooks in Slovak and Czech language
Villanueva & Gisbert: Medicina Legal y Toxicología
Primarily Simpson´s Forensic Medicine. Knight´s Forensic Pathology for those more ambitious
Knight's Forensic Pathology by Pekka Saukko, Bernard Knight; Handbook of Forensic Medicine 1st Edition by Burkhard Madea (Editor); in Latvian – Prof O Teteris—Tiesu Medicīnas Esence
Madea B. Rechtsmedizin. 3rd ed.Springer
Teresiński (ed.) Medycyna Sądowa
Simpson's Irish Edition
Knight B, Saukko P. Knight`s Forensic Pathology, Third Edition 2004. Mason JK. The Pathology of Trauma, Edward Arnold Ltd, 1993. Kumar V, Abbas KA, Fausto N. Robbins and Cotran Pathologic Basis of Disease (Elsevier Saunders), 2005. Henssge C, Knight B, Krompecher T, Madea B, L. Nokes – The estimation of the time since death in the early postmortem period, London, Sydney, Auckland, Co-published in the USA by Oxford University Press, Inc., New York, 1995. Madea B, Musshoff F, Tag B, (2011) Kurzlehrbuch Rechtsmedizin, Huber, Bern. Madea B, (2019): Die ärztliche Leichenschau, 4th ed Springer, Heidelberg. Madea B, Prangenberg J, Ulbricht J, Doberentz E, (2022): Feststellung der Todesursache, Lehmanns Media, Berlin
Saukko P, Knight B (2004). Knight's Forensic Pathology (3rd ed.). London: Edward Arnold (Publishers). Vieira DN, Quintero A: Aspectos práticos da avaliação do dano corporal em Direito Civil. Colecção Biblioteca Seguros, Imprensa da Universidade de Coimbra, 2008. Gisbert Calabuig – Medicina Legal y Toxicología; Enrique Villanueva Cañadas; 6ª Edición 2005. Masson. Payne-James J, Byard RW, Corey TS, Henderson C. Encyclopedia of Forensic and Legal Medicine. London: Elsevier Academic Press; 2005
Dettmeyer RB, Verhoff MA, SSchütz AF. Forensic Medicine: Fundamentals and Perspectives. Berlin: Springer Verlag, 2013. Payne-James JJ, Byard RW. Encyclopedia of Forensic and Legal Medicine. 2nd edition. Elsevier, 2016. Vij K. Textbook of Forensic Medicine and Toxicology: Principles and Practice. 5th edition. New Delhi: Elsevier, 2011
Simpson's Forensic Medicine, Payne-James J, Jones R, 14th Ed., CRC Press, 2019.Reading List Knight's Forensic Pathology, Saukko P, Knight B, 4th Ed., CRC Press, 2016. Clinical Forensic Medicine, McLay W, 3rd Ed., Cambridge University Press, 2009. http://www.forensicmed.co.uk/
Bulgarian textbook of forensic medicine and deontology, Simpson's Forensic Medicine. Payne-James, Jones R, Karch SB, Manlove J. Knight’s Forensic Pathology, Saukko P, Knight,B. Oxford Handbook of Forensic Medicine – Wyatt J, Squires T, Norfolk G, Payne-James J. Madea B, (2022): Handbook of Forensic Medicine, 3 Vol., Wiley, Chichester, UK, Forensic Pathology by Di Maio VJM, Di Maio DJ
Collège des Enseignants de Médecine Légale
Collège de Médecine Légale (Elsevier Masson)
Simpson´s Forensic Medicine Knight´s forensic pathology
Elsevier Masson—Collège Médecine Légale
Nouvelle édition 2022 de Médecine légale—Médecine du Travail, sous l’égide du Collège national des Enseignants de Médecine Légale, du Collège des Enseignants Hospitalo-Universitaires de Médecine et Santé au Travail et de la Société Française de Médecine Légale, dans la collection des Référentiels des Collèges
Medicina Legale, Silingardi, De Leo, Introna. Di Vella, Campobasso, 2023, Ed. Idelson-Gnocchi
Encyclopedia of Forensic and Legal Medicine
Dettmeyer RB, Verhoff MA, Schütz HF. Forensic Medicine Fundamentals and Perspectives Springer ISBN 978–3-642–38817-0 ISBN 978–3-642–38818-7 (eBook) Jason Payne-James ed.: Simson's Forensic Medicine 13th Edition, 2011 Hodder Arnold an Hachette UK Company ISBN-13 978 0 340 986 035 Lecture Notes of Forensic Medicine (Ed.: P. Sótonyi, E. Keller), Semmelweis Publisher, 2008. ISBN 978 963 9656 92 5 P. Sótonyi: Igazságügyi Orvostan, Semmelweis Kiadó, Budapest, 2010

## Discussion

This survey has provided a previously unanalysed insight into the teaching of undergraduate forensic medicine across many European countries which are part of the ECLM.

The survey response rate is well within the response rate expected for such electronic surveys. The response rate (45%) was slightly higher than that of average response rates (44.1%) based on meta-analysis of online surveys [6]. The responses from respondents were detailed allowing a gross picture of the situation in European countries. All data from responses received were included and although this might have some effect on the data regarding subjects taught and teaching methods used (eg by overemphasising some subjects and teaching methods), it was considered that each respondent would be responding to the questions based on their own personal experience and thus would have validity. The survey non-responses also reinforce the difficulties of inaccuracies and inactive email contacts within the ECLM contact database, despite considerable effort by the ECLM Executive Committee to ensure that it is as up to date as possible.

In some questions, intra-country inconsistency when considering numbers of medical schools and graduates was observed, but this is understandable when taking into consideration the nature of the particular questions. For example,the differences in responses to some questions from forensic practitioners in the same country show that data which might be expected to be consistent and easily found is not always the case. Notably the different responses from practitioners in the same countries about the number of medical schools and the number of doctors trained show that accurate data are not always readily available or perhaps known. Discrepancy in responses may also be related to different interpretations of the question. One example is in the case of the responses from Italy, where it is likely that the high number of schools in one of the answers (see Table 2) includes not only medical training but other training such as dentistry, as in Italy legal medicine is a compulsory subject in healthcare training including nursing, physical therapy and occupational therapy.

Some answers may have been guessed or estimated by respondents. In addition, some of the answers to the English-language survey show that it is not always possible to frame a question to ensure that a consistent response is received, this is despite the questions having been agreed by consensus by a multilingual group of ECLM Executive Committee members, most of whom do not have English as their first language, although the working language is English.

With regard to the main focus of the survey, it is clear that the majority of respondent countries have forensic medicine as a compulsory part of the undergraduate medical curriculum. The population covered by those who have responded is just over half a billion people. The United Kingdom and Belgium responses were notable as they did not have systematic teaching in forensic medicine in their universities’ medical curricula.

The survey showed that there is a strong consistency in the forensic medicine subjects taught in European countries with a wide variety of training methodology, but a heavy emphasis on lectures. Practical demonstrations and attendance at forensic autopsies were popular, but it is disappointing that attendance at clinical forensic examinations was the least used, as for most doctors it is the clinical aspects of forensic medicine that they are most likely to encounter in routine medical practice. Reasons for this may include concerns about medical or legal confidentiality.

From the ECLM perspective it is of interest that almost a third of respondents had no awareness of the Perugia Document [4]. What is reassuring is that forensic medicine has considerable profile in undergraduate teaching in most European countries. It appears that there is an overwhelming recognition in ‘Europe’ [however defined] of the need for undergraduate forensic medicine teaching, and as such this should provide future clear opportunities for a pan-European collaborative approach in undergraduate training.

The data from this survey provide a baseline which should assist in developing a strategy to harmonise forensic medicine undergraduate training in member countries of the ECLM. There have been a number of initiatives involved in the teaching of forensic medicine to undergraduates including a web-based e-learning programmes for training external post mortem examination [7], the use of special study modules in forensic & legal medicine as optional additions to the medical curriculum [8], the introduction of objective structured clinical examinations on external post mortem examination [9], the use of computer-based learning games [10] and the use of educational video on forensic autopsy [11]. These initiatives all indicate the potential and opportunity for creating multi-modal teaching in forensic medicine for a contemporaneous cohort of undergraduates. The ECLM has a strong history of producing consensus documents that may be used as a basis for training, development and practice in European countries. Examples of previous publications include on-site inspection forms for forensic pathology, anthropology, odontology, genetics, entomology and toxicology for forensic and medico-legal scene and corpse investigation [12, 13], accreditation of forensic pathology services in Europe [14], and elder abuse [15].

The findings of the survey show that in order to establish further and guarantee the position of the forensic medicine in the medical curriculum, the ECLM delegates need to determine for their own country: i) key contacts in medical education responsible for the medical curriculum; ii) accurate numbers of undergraduate medical trainees; and c) accurate contact details for all medical schools & colleges in order to ensure the appropriate dissemination of relevant knowledge.

Finally, it is clear that there is a great need to update the ‘Perugia document’ which is now over three decades old [3]) or to develop a new advanced and modernised document to replace it, and harmonise forensic medicine undergraduate education in European countries.

With the survey presented here, the ECLM is now in a good position to establish a pan-European working group to coordinate a consensus document identifying an appropriate and modern core undergraduate forensic medicine curriculum that can be presented to the medical education authorities in each country, and which can be adapted for local requirements, based on available personnel, the forensic medicine structure in the country, and most importantly, the needs of the local population. The ECLM and the legal and forensic medicine community should seize the opportunity to raise such issues with politicians, stakeholders and opinion leaders.

## Appendix 1 Introductory email sent to ECLM database (as at 11^th^ June 2023)

Dear colleague,

The European Council of Legal Medicine (www.eclm.eu) is undertaking a survey of all countries within its membership to determine what undergraduate teaching of forensic & legal medicine takes place in each country. You have been asked to complete this survey as you are noted to be either a delegate, deputy, observer to the ECLM or a representative of your country's National Authority for forensic medicine. If you have received another request, please only complete the survey once.

The survey is an online one that should take no more than ~ 10–15 min and we would be very grateful if you would complete the survey as soon as possible. If you feel you are unable to complete the survey for any reason please let us know.

## Appendix 2 List of questions asked in the ECLM undergraduate forensic medicine survey

1. In which country do you work?
2. What is your job title?
3. How many medical schools are there in your country?
4. How many medical doctors complete their training in your country each year?
5. Is forensic medicine (sometimes known as legal medicine, forensic & legal medicine, or legal & forensic medicine) a compulsory part of undergraduate medical training/curriculum in your country? [Yes/No]
6. If Yes to question 5—please answer subsequent questions, if No to 5 – thank you for completing the survey
7. If Yes to question 5 – how many hours training are allotted to the forensic medicine module? [Free text]
8. If Yes to question 5 – are there differences between different medical schools? [Free text]
9. During which year / semester of the studies is forensic medicine taught in your country? If it varies between Universities, please give examples and range [Free text].
10. Which of the following subjects are taught as part of forensic medicine to undergraduates[Yes/No responses – tick all that apply]?:
  - Autopsy demonstration
  - Classification of injury
  - Complaints of torture or cruel, inhuman and degrading treatment
  - Confidentiality
  - Consent
  - Differences between natural death and unnatural death
  - Ethics & Medical Law
  - Documentation and interpretation of injury
  - Examination of complainants of physical assault
  - Examination of complainants of sexual assault
  - Examination of those arrested for driving offences
  - Forensic genetics
  - Forensic toxicology
  - Giving live evidence in court
  - Legal duties of doctors
  - Medical negligence
  - Overview of the role of the forensic pathologist
  - Overview of the role of the forensic physician [clinical forensic medicine]
  - Police process for crime
  - Professional and ethical duties of doctors
  - Role of the doctor after a patient dies including writing a Medical Certificate of Cause of Death
  - The medicolegal investigation of death
  - Writing a statement for court
  - Other [free text]
11. Is the teaching of the forensic medicine module done by [Yes/No responses – tick all that apply]:
  - Lectures
  - Workshops
  - Small group teaching
  - Practical demonstrations
  - Attendance at clinical forensic examinations
  - Attendance at forensic autopsies
  - Other [free text]
12. Is completion of the forensic medicine module teaching confirmed by [Yes/No to all that apply]:
  - Written examination
  - MCQ examination
  - Oral examination
  - Informal assessment
  - Formal assessment
13. If yes to question 9, is the examination graded? [Yes/No]
  - by numbers (e.g. 1–5)
  - by percent
  - by pass/fail
14. What are the materials recommended/provided / demanded for the course / examination [free text and Yes/No responses]?
  - Text book(s), if yes, give the name of the book(s) [free text]
  - Notes in the form of Powerpoint or similar
  - Scientific articles
  - Popular articles
  - Videos
  - Others, please specify
15. Would you be happy to address further specific questions once the survey results have been compiled? Yes/No]
16. If Yes to 15, please provide your full name and contact details [free text]
